# Long-Acting Injectable Antipsychotics for First-Episode Schizophrenia: The Pros and Cons

**DOI:** 10.1155/2012/560836

**Published:** 2012-08-14

**Authors:** Borah Kim, Sang-Hyuk Lee, Yen Kuang Yang, Jong-Il Park, Young-Chul Chung

**Affiliations:** ^1^Department of Psychiatry, CHA Bundang Medical Center, CHA University, 59 Yatap-ro, Bundang-gu, Seongnam-si 463-712, Gyeonggi-do, Republic of Korea; ^2^Department of Psychiatry, National Cheng Kung University Hospital, College of Medicine, National Cheng Kung University, 138 Sheng Li Road, Tainan 70428, Taiwan; ^3^Department of Psychiatry, Chonbuk National University Medical School, 567 Baekje-daero, Deokjin-gu, Jeonju-si 561-756, Jeollabuk-do, Republic of Korea; ^4^Research Institute of Clinical Medicine of Chonbuk National University, Biomedical Research Institute of Chonbuk National University Hospital, 20 Geonjiro, Deokjin-gu, Jeonju-si 561-712, Jeollabuk-do, Republic of Korea

## Abstract

Clinical and psychosocial deterioration associated with schizophrenia occurs within the first few years following the onset of the illness. Therefore, to improve the long-term prognosis, it is important to provide schizophrenia patients with intensive treatment following their first episode. Relapse is highly associated with partial medication adherence or nonadherence in patients with first-episode schizophrenia. Recent studies suggest that long-acting injectable (LAI) antipsychotics compared with oral antipsychotics are more effective for medication adherence and relapse prevention. Moreover, some clinical guidelines for the treatment of schizophrenia suggested that LAI antipsychotics should be considered when patients are nonadherent “at any stage.” Decreased compliance is a common cause of relapse during the initial stages of the disease. Therefore, LAI antipsychotics should be highly considered when treating patients with first-episode schizophrenia. In the present paper, clinical trial data and current guidelines on the use of LAI antipsychotics for first-episode schizophrenia are discussed as well as the pros and cons of this treatment option.

## 1. Introduction

Schizophrenia is a chronic disorder characterized by periods of illness alternating with periods of full or partial remission. Previous studies [1, 2] suggest that schizophrenia is a neurodegenerative disease associated with frequent relapses. This alternating nature of the illness causes neurotoxicity in the brain, thereby resulting in structural abnormalities, including ventricular enlargement and cortical atrophy. Recent evidence further suggests that progressive structural changes in the brain occur within the initial years following a diagnosis [3–5]. Moreover, with each subsequent relapse after the first episode, it usually takes longer time to reach remission [6]. The primary clinical and psychosocial deterioration associated with schizophrenia occurs within the first 5 years following the onset of the illness, called the critical period [7, 8]. Therefore, it is important to provide intensive biopsychosocial interventions during the critical period in an effort to improve the long-term prognosis.

The primary goal of treatment during the critical period is to prevent a subsequent relapse and to restore socio-occupational functioning to the premorbid level. The relapse rate in patients with first-episode schizophrenia is relatively low during the first year of the illness but substantially rises to rates of 53.7% and 74%–81.9% after 2 and 5 years, respectively [9, 10]. The most common cause of relapse in patients with schizophrenia is a lack of adherence to oral medication [11, 12]. The discontinuation of antipsychotics in patients with first-episode schizophrenia or schizoaffective disorder increases the risk of relapse by approximately five times [9]. The rate of medication discontinuation in individuals with first-episode psychosis ranges from 26% [13] to 44% [14] during the first year. Coldham et al. [15] reported a 59% rate of poor adherence (39% nonadherent and 20% inadequately adherent) within the year after the first episode. None of the interventions currently used to improve adherence have been completely reliable in the treatment of schizophrenia. Long-acting injectable (LAI) antipsychotics, however, may improve medication adherence and possibly reduce relapse in patients with schizophrenia [16]. The Texas Medication Algorithm Project Antipsychotic Algorithm for schizophrenia [17] recommended that clinicians should assess contributing factors and consider LAI antipsychotic preparations in patients who are inadequately adherent at any stage. Previous studies have suggested that LAI antipsychotics may be more effective for maintaining medication adherence [18] and preventing relapse [19] in first-episode schizophrenia compared with oral antipsychotics. Clinically, however, the majority of psychiatrists use LAI antipsychotics very conservatively [20, 21]. Moreover, very few psychiatrists offer LAI antipsychotics after a patient's first psychotic episode [22]. Recently, Osborne et al. (2012) reported that society associates a higher utility with increasing time between injections, with 4-weekly and 3-monthly administration of an antipsychotic LAI representing an advance over 2-weekly administration in the health-related quality of life (HRQoL) of patients with schizophrenia [23]. They discussed that participants from the general population preferred less frequent injections given the psychological stress and pain associated with injections as well as the burden related to the requirement of travel to outpatient clinics for their administration. In this regard, currently available option of LAI antipsychotic with one month injection interval would add substantial value to the improvement of quality of life in patients with schizophrenia and their family members. Optimal therapeutic outcomes associated with first-episode psychosis are often compromised by early treatment discontinuation and poor treatment adherence; therefore, LAI antipsychotics should be more actively considered with first-episode schizophrenia.

The present paper reviews the relevant literature including clinical drug trials, survey studies, and clinical guidelines on the use of LAI antipsychotics in patients with first-episode or recent-onset schizophrenia. Moreover, the pros and cons of LAI antipsychotic used with first-episode schizophrenia are discussed.

## 2. Overview of Clinical Studies on the Effectiveness of Long-Acting Injectable Risperidone Treatment for First-Episode or Recent-Onset Schizophrenia (Table 1)

In a study by Parellada et al. [24], 382 patients received long-acting injectable risperidone (RLAI) treatment during the early stages of their disease (i.e., within 3 years of diagnosis). The study, conducted in Europe, evaluated the efficacy and safety of RLAI. It was an open-label, nonrandomized, and single-arm, multicenter study that consisted of a 6-month treatment period. Significant improvements in total Positive and Negative Syndrome Scale (PANSS) scores were noted at the first visit and continued to improve through the end of the study. At the end of the study, 148 patients (40%) displayed an improvement in total PANSS and subscale scores. Functioning is also improved from baseline to endpoint, with a mean Global Assessment of Functioning (GAF) score of 57.6 (±6.5) at baseline and a mean GAF score of 65.3 (±18.3) at the end of the study. Adverse events were reported by 263 patients (69%), including extrapyramidal symptoms. These symptoms, however, improved significantly over the 6-month treatment period. Parellada [25] also reported that individuals treated with RLAI, including patients with first-episode schizophrenia, demonstrated improvement in symptom severity and compliance as well as a reduction in the rate of relapse and a favorable tolerability profile.

According to a study that compared RLAI and oral risperidone for the treatment of first-episode schizophrenia [19], significant improvements in PANSS, GAF, and Clinical Global Impression (CGI) were noted in the RLAI group compared with the oral risperidone group. No significant differences in extrapyramidal symptoms or frequency of prolactin-related adverse effects were noted in either group. Moreover, medication adherence was higher, and the relapse rates at 1 and 2 years were significantly lower in the RLAI group. The oral risperidone group showed significantly greater nonadherence. In the study, the amount of time to nonadherence predicted the relapse in patients with first-episode schizophrenia. The authors proposed that RLAI may be effective in preventing relapse through maintaining medication adherence. The authors [30] also emphasized that psychosocial interventions for relapse prevention may be effective for maintaining medication compliance in patients with schizophrenia who receive RLAI.

A single-site open-label study of 50 patients with first-episode schizophrenia who were treated with RLAI [26] found that 32 patients (64%) achieved remission. Of the 32 patients, 31 (97%) maintained remission throughout the study. The remission and nonremission groups were compared based on clinical, functional, and quality-of-life outcome measures. The remission group showed significantly greater improvements on the CGI-Severity (CGI-S) scale and PANSS total score and also displayed improvements in extrapyramidal symptoms. Remission patients received lower doses of RLAI and showed greater improvement in social functioning compared with the nonremission group in the study. Multivariate level analyses showed that the chance of remission increased in females. Early symptom improvement was also significantly associated with remission. Moreover, less decline in the PANSS score was associated with a reduced likelihood of remission. The remission rate in this study compared favorably with that reported in previously published studies could be benefit of assured antipsychotic delivery and better adherence of treatment of RLAI. In another study conducted at the same site [26], compared with patients treated with oral risperidone or haloperidol, RLAI-treated patients had significantly fewer all-cause discontinuations (26% versus 70% at 24 months), greater symptom reduction according to PANSS total score (−39.7 versus −25.7), higher remission rates (64% versus 40%), lower relapse rates (9.3% versus 42%), and lower extrapyramidal symptoms [27].

A randomized controlled trial reported on acceptance and initial adherence outcomes with RLAI treatment in patients with first-episode schizophrenia [18]. According to the results, 73% of subjects (19 out of 26) randomly assigned to receive RLAI accepted. Individuals who took RLAI were significantly more likely to remain adherent at 12 weeks compared with patients treated with oral antipsychotics. The authors suggested that better adherence was achieved in patients who received RLAI through two possible explanations. One is that starting treatment with RLAI might have direct adherence benefits in preventing or delaying nonadherence in some individuals. The other one is that refusal of the RLAI recommendation is a sign that the patient plans to stop oral medication in the very near future, which means that the more important thing is not so much the RLAI itself but the will to stay on treatment with any antipsychotics. The researchers anticipated that some patients would refuse the RLAI recommendation; however, they found that about 73% of subjects accepted the recommendation for treatment with RLAI. This suggests the feasibility and acceptability of LAI antipsychotics as a treatment strategy during the early stages of schizophrenia.

According to an open-label noncomparative study of recent-onset schizophrenia [28], patients showed good progress as indicated by symptom reduction, improved functional outcomes, and improved health-related quality of life. Rabinowitz et al. [31] also demonstrated that improved premorbid functioning in patients with schizophrenia is predictive of increased treatment response with RLAI as measured by clinical rating scales of symptoms and functioning, health care-related quality of life, and remission.

A multicenter, nonintervention, observational study on the clinical effectiveness of RLAI administration was published in 2011 [29]. Treatment with RLAI for 12 months during the early stages of schizophrenia was associated with significant improvements in clinical and functional outcomes in patients. This investigation suggested the effectiveness of RLAI for treatment of patients early in the course of schizophrenia in a similar vein to previous studies [19, 26] with the result from relatively large subjects (*n* = 105).

A recent study by Bartzokis et al. [32] compared RLAI and oral risperidone treatment in subjects with first-episode schizophrenia. The study focused on changes in frontal lobe myelination and cognitive functioning. White matter (WM) volume remained stable in the RLAI group and decreased significantly in the oral risperidone group, resulting in a significant difference on the effects of treatment. RLAI seems to promote myelination and stabilizes frontal lobe WM volume compared with oral risperidone. Moreover, the changes in frontal lobe WM volume were positively associated with higher-order executive functioning, working memory, and mental flexibility. No significant volume changes were noted in the frontal lobe. The authors suggested that the changes in WM and gray matter (GM) represent a myelination-driven shift of the GM/WM boundary into or out of the cortex. The myelination trajectory was significantly quadratic (inverted U) and peaked at 1 year of antipsychotic treatment. This was followed by a premature decline compared with healthy subjects who do not decline until after the fifth decade of life. The nonsignificant increase in WM volume observed with RLAI suggests that the trajectory defined by oral antipsychotic treatment may be modifiable with RLAI. Therefore, the consistent medication levels that are achieved with RLAI may result in a higher WM volume, which may subsequently impact cognitive performance.

Although favorable results of LAI antipsychotics have been reported for patients with first-episode or recent-onset schizophrenia, the effectiveness of LAI treatment for patients with chronic schizophrenia remains controversial. A meta-analysis of depot antipsychotics was conducted in 2001 [33]. This report suggested that depot antipsychotics were statistically better for global improvements compared with oral antipsychotics. However, relapse, attrition, and adverse effects were not significantly different. In addition, a recent long-term randomized controlled trial that included patients with unstable schizophrenia [34] demonstrated that RLAI was not superior to oral treatment in terms of duration of adherence, time to rehospitalization, clinical symptoms, or improvement in functional outcome. Moreover, this study reported that RLAI treatment was associated with more local injectionsite and extrapyramidal adverse effects. On the other hand, the recent meta-analysis [35] comparing LAI with oral antipsychotics showed that a reduced risk for relapse was associated with LAI over oral antipsychotics. To clarify clinical issue related to the use of LAI antipsychotics in first-episode or recent-onset schizophrenia, further studies, especially randomized controlled trials, are warranted especially with regard to the effectiveness on relapse or rehospitalization.

## 3. The Pros and Cons of LAI Antipsychotics (Table 2)

The general attitude of psychiatrists toward depot antipsychotics is negative. Depot antipsychotics are considered old-fashioned, stigmatized, and less acceptable to patients. Many psychiatrists stated that first-generation depots are avoided because of the threat of extrapyramidal side effects, whereas second-generation LAI drugs are associated with high treatment costs [20]. It is of interest to see two opposing opinions about the use of LAI drugs in first episode of psychosis in the recent surveys. Over half of the psychiatrists in the UK who participated in a survey agreed that LAI drugs can be used in patients with first-episode psychosis [21]. In contrast, Heres et al. [20] reported that the majority of psychiatrists (64–71%) applied the “no depot in first-episode psychosis” rule. They also recently investigated factors associated with psychiatrist's negative attitude toward offering depot treatment to first-episode patients and found that three factors, limited availability of different second-generation antipsychotic depot drugs, the frequent rejection of the depot offer by the patients, and the patients' skepticism based on the lack in experience of a relapse, were of marked influence [36]. In a study conducted in Switzerland, fewer than 10% of psychiatrists offered depot treatments in response to the first psychotic episode [22]. Given that psychiatrists are relatively conservative in offering information about depot antipsychotics [22] and that some patients feel positively toward treatment with depot medication [37, 38], more patients with first-episode psychosis may accept LAI medication if psychiatrists provided them with adequate information. The best rationale for using LAI antipsychotics in first episode of psychosis comes from the fact that frequent relapses occur during first few years of the illness, and there is evidence for decreased rate of relapse with LAI medication compared to oral antipsychotic drug in first-episode schizophrenia [19, 30, 39]. Another advantage may be that LAI antipsychotics can improve patient's quality-of-life overtime with more possibilities to meet friends and family, to live a more stable and independent life, outside the psychiatric hospital [40]. Patients with schizophrenia are more sensitive to adverse drug effects during the first few years of the illness [41–43]; therefore, the low incidence of adverse events caused by low variation in peak and trough levels of LAI antipsychotics may have additional benefits for pharmacological compliance during the critical period. Some people argued that the best time to prescribe LAI antipsychotics is just prior to discharge. One argument against the use of LAI antipsychotics for the treatment of first-episode psychosis may be related to the uncertainty of the diagnosis. If brief psychotic disorder, not otherwise specified (NOS) psychotic disorder, or schizophreniform disorder is suspected, the recommended duration of treatment should be much shorter, and a greater portion of patients may have a chance of recovery than in case of schizophrenia. For such cases, the patient's autonomy is even more important in the process of treatment decision. Because of negative association of injection with coercion, recommending LAI antipsychotics by treating doctors would hamper therapeutic relationship. Moreover, it would discourage patient's motive to recover because of the general perception that injection treatment option usually means severe condition of the illness. Moreover, due to the difficulty of adjusting the dose of LAI drugs quickly in response to side effects, treatment compliance may be negatively affected during the critical period. To improve the conservative attitude of psychiatrists with respect to LAI antipsychotic treatment for patients with first-episode schizophrenia, several issues must be tackled. First, the development of more accurate subjective or objective measures that predict or detect drug compliance in patients with first-episode schizophrenia should be pursued. Second, more diverse second-generation depot formulations should be available for the current clinical practice, like paliperidone palmitate, olanzapine pamoate, aripiprazole, or iloperidone depot. The development of completely different formulae, such as a patch drug containing olanzapine [44] or risperidone [45], would have wide applicability for patients with first-episode schizophrenia because one of the primary reasons why individuals reject LAI drugs is the fear of needles. Third is about using financial incentives may improve adherence to antipsychotic maintenance medication [46]. This applies not only to first-episode schizophrenia but also to multiple-episode chronic schizophrenia and raises its ethical issues [47]. In Japan, counseling and management fees have been used for depot antipsychotics since 1990 to promote the use of depot antipsychotics for schizophrenia (personal communication). It remains to be seen how financial incentives will unfold especially in relation to first-episode schizophrenia.

## 4. Guidelines for the Treatment of First-Episode Schizophrenia with LAI Antipsychotics

According to the American Psychiatric Association [48], LAI antipsychotic medication is recommended for patients with recurrent relapses related to partial or full nonadherence. Also, the Canadian clinical practice [49] recommends LAI formulations to reduce nonadherence in multiple-episode patients or patients with persistent positive symptoms. The International Psychopharmacology Algorithm Projects (IPAPs) schizophrenia algorithm (http://www.ipap.org/schiz/) suggested that depot antipsychotics were recommended in patients with partial or complete noncompliance. However, there was no mention of LAI for the treatment of first-episode schizophrenia. These guidelines, therefore, limit the use of LAI to patients characterized as multiple episodes or noncompliance.

However, there have been subtle changes in more recent guidelines. For example, the procedural manual by the Texas Medication Algorithm Project [17] recommends that the clinicians assess contributing factors and consider LAI antipsychotics in patients who are inadequately adherent “at any stage.” It means that LAI could be used even in first-episode schizophrenia if the patients are not enough adherent to their medications. Similarly, in 2009, the National Institute for Health and Clinical Excellence (NICE) guidelines regarding schizophrenia (http://www.nice.org.uk/CG82) stated that clinicians should consider offering depot/LAI antipsychotic medications to patients with schizophrenia who would *“prefer such treatment after an acute episode and where avoiding covert non-adherence to antipsychotic medication is a clinical priority”* within the treatment plan. Kane and Garcia-Ribera [50] also mentioned that LAI can be indicated to *“any schizophrenia”* patients requiring long-term treatment, nonadherent, or having risk of relapse. They further suggested that even if patients refuse this option, it would be better to help them understand the potential advantages. On the other hand, recent guidelines from the British Association for Psychopharmacology [51] described that the place of antipsychotic depot/long-acting injections for first-episode schizophrenia remains uncertain on account of the absence of long-term data comparing LAI with oral antipsychotics after first-episode schizophrenia.

It is still true that LAI has a conservative position in treating first-episode schizophrenia according to the majority of the current guidelines. Nevertheless, considering the results from LAI studies in first-episode psychosis previously, future guidelines might be needed to update a treatment option to recommend LAI antipsychotics for any patients with schizophrenia who showed poor adherence attitude and behavior including first-episode schizophrenia.

## 5. Conclusions

With the availability of the different second-generation LAI antipsychotics, there are improved treatment options for schizophrenia in terms of duration of action and side effects. Psychiatrists, however, seem to conservatively use depot formulations and mostly introduce them after several episodes. The acceptability of prescribing LAI antipsychotics to patients with first-episode psychosis is currently under debate. Many clinical and technical issues should be addressed to encourage increased acceptability of LAI antipsychotics for the treatment of patients with first-episode schizophrenia. Nevertheless, given that low compliance is a frequent cause of relapse in the early course of schizophrenia, more active consideration of LAI drugs should be encouraged, and patients should be informed about the different types of medication that are available during the early stages of the illness. Further studies, especially randomized controlled trials, are urgently needed to clarify the advantages of second-generation LAI antipsychotics in patients with first-episode schizophrenia.

## Figures and Tables

**Table 1:** Overview of clinical studies on the effectiveness of long-acting injectable antipsychotics for the treatment of first-episode or recent-onset schizophrenia.

Study	No. of patients (female/male)	No. of previous episodes	Duration of illness	Study duration	Design	Dosage	Reduction of total PANSS (%)	Reduction of CGI-S (%)	Functional improvement (%)	Tolerability	Adherence	Long-term outcome
Parellada et al. [24]	382 (117/265)	ns	1.5 (1.1) yr	6 mo	Open, 1-arm, mc	25–50 mg	18.3%	ns	GAF 13.4%	ESRS 53.8%↓ PRL-related 0.3% wt gain 4%	ns	ns

Kim et al. [19]	RLAI: 22 (14/8) Oral: 28 (17/11)	1	1.5 (1.5) yr	2 yr	Open, C	25–50 mg	RLAI: 10.0% Oral: 2.0%	RLAI: 10.0 Oral: 2.5	GAF RLAI: 26.9% Oral: 0.5%	ns	RLAI: GA (>70%) 68% Oral: GA 32%	2-yr relapse RLAI: 23% Oral: 75%

Emsley et al. [26]	50 (18/32)	1	≤1 yr	2 yr	Open, 1-arm	25–50 mg	Remission: 45.0% No remission: 29.7%	Remission: 73.1% No remission: 34.4%	SOFAS remission: 85.2% No remission: 43.2%	ESRS 53.3%↓ in remission; 55.0%↑ in no remission	ns	2-yr remission 62%

Emsley et al. [27]	RLAI: 50 (18/32) Oral: 47 (20/27)	≤2 adm	≤ 1yr	2 yr	Post hoc comparison RLAI: Open, 1-arm Oral: R, C (ris versus hal)	25–50 mg	RLAI: 44.0% Oral: 28.8%	ns	ns	Total maximum changes of *ESRS * RLAI: 1.40 (2.60) Oral: ris 5.61 (5.22) hal 9.04 (6.21)	ns	2-yr remission RLAI: 64.0% Oral: 40.4%

Weiden et al. [18]	RLAI: 19 Oral: 11	1	≤16 wk of lifetime AP exposure	12 wk	Open, R, C	25–37.5 mg	ns	ns	ns	ns	RLAI: 89% Oral: 59%	ns

Napryeyenko et al. [28]	294 (116/178)	2.4 (0.7)	≤2 yr	26 wk	Open, 1-arm, mc	25–50 mg	18.6%	20.5%	GAF 16.9%	EPS 5.6% PRL-related 4.3% wt gain 3.0%	NC, *n* = 3	1-yr remission 59.5%

Dubois et al. [29]	105 (79/26)	<4	3.0 (3.92) yr	12 mo	Open, 1-arm, mc	25–50 mg	ns	43.4%	GAF 71.1%	EPS 3.8% PRL-related 9.5% wt gain 18.1%	>80%	1-yr remission 34.1%

PANSS: Positive and Negative Syndrome scale; CGI-S: Clinical Global Impression-Severity scale; GAF: Global Assessment of Functioning; SOFAS: Social Occupational Functioning Assessment scale; ESRS: Extrapyramidal Symptom Rating scale.  adm: hospital admissions for psychosis; AP: antipsychotics; C: controlled study; EPS: extrapyramidal symptoms; GA: good adherence (>70%) group; hal: haloperidol; mc: multicenter; mo: month; NC: noncompliance; ns: not specified; open: open-label; oral: oral risperidone; PRL: prolactin; pts: patients; R: randomized; ris: risperidone; RLAI: long-acting injectable risperidone; wk: week; wt: weight; yr: year; 1-arm: single arm.

**Table 2:** The pros and the cons of using long-acting injectable (LAI) antipsychotics for the treatment of first-episode schizophrenia.

Pros	Cons
High relapse caused by poor compliance could be prevented	Because of uncertainty of diagnosis for those in first-episode psychosis, prescribing LAI drugs may be stigmatizing and may hamper therapeutic relationships

Some high-functioning individuals may prefer depot formulations	Discourage patient's motive to recover because of the general perception that an injectable treatment means a more severe condition with respect to the illness

Favorable side effect profile due to low variation in the peak and trough levels would have positive effects on drug compliance	For those with first-episode schizophrenia showing a positive outcome, the goal of treatment is to gradually reduce the dosage of antipsychotics, which does not fit the traditional goals of LAI drugs

Best time to prescribe LAI drugs may be just before discharge	It is difficult to adjust the dosage of LAI drugs quickly in response to side effects; therefore, LAI treatment may negatively affect subsequent treatment compliance during the critical period
